# Lipo-PGE1 suppresses collagen production in human dermal fibroblasts via the ERK/Ets-1 signaling pathway

**DOI:** 10.1371/journal.pone.0179614

**Published:** 2017-06-23

**Authors:** Yoolhee Yang, Hee Jung Kim, Kyong-Je Woo, Daeho Cho, Sa Ik Bang

**Affiliations:** 1 Department of Plastic Surgery, Samsung Medical Center, Sungkyunkwan University School of Medicine, Seoul, Korea; 2 Institute of Women’s Life Medical Science, Department of Obstetrics and Gynecology, Yonsei University College of Medicine, Seoul, Korea; 3 Department of Life Systems, Sookmyung Women’s University, Seoul, Korea; Oklahoma State University Center for Health Sciences, UNITED STATES

## Abstract

Dysregulation of collagen production contributes to various pathological processes, including tissue fibrosis as well as impaired wound healing. Lipo-prostaglandin E1 (Lipo-PGE1), a lipid microsphere-incorporated prostaglandin E1, is used as a vasodilator for the treatment of peripheral vascular diseases. Lipo-PGE1 was recently shown to enhance human dermal fibroblast (HDF) migration and *in vivo* wound healing. No published study has characterized the role of Lipo-PGE1 in collagen regulation in HDFs. Here, we investigated the cellular signaling mechanism by which Lipo-PGE1 regulates collagen in HDFs. Collagen production was evaluated by the Sircol collagen assay, Western blot analysis of type I collagen and real time PCR. Unexpectedly, Lipo-PGE1 decreased mRNA expression of collagen 1A1, 1A2, and 3A1. Lipo-PGE1 markedly inhibited type I collagen and total soluble collagen production. In addition, Lipo-PGE1 inhibited transforming growth factor-β-induced collagen expression via Smad2 phosphorylation. To further investigate whether extracellular signal-regulated kinase (ERK)/Ets-1 signaling, a crucial pathway in collagen regulation, is involved in Lipo-PGE1-inhibited collagen production, cells were pretreated with an ERK-specific inhibitor, PD98059, prior to the addition of Lipo-PGE1. Lipo-PGE1-inhibited collagen mRNA expression and total soluble collagen production were recovered by pretreatment with PD98059. Moreover, Lipo-PGE1 directly induced the phosphorylation of ERK. Furthermore, silencing of Ets-1 recovered Lipo-PGE1-inhibited collagen production and PD98059 blocked Lipo-PGE1-enhanced Ets-1 expression. The present study reveals an important role for Lipo-PGE1 as a negative regulator of collagen gene expression and production via ERK/Ets-1 signaling. These results suggest that Lipo-PGE1 could potentially be a therapeutic target in diseases with deregulated collagen turnover.

## Introduction

Human dermal fibroblasts (HDFs), one of the main cell types in the skin, play a key role in the pathophysiology of fibrotic diseases as well as in cutaneous wound repair [1, 2]. Dermal fibroblasts facilitate wound healing through enhancement of their migration, proliferation, and collagen production. Collagen, one of the most plentiful and ubiquitous proteins in the human body, is a major component of extracellular connective tissue matrix (ECM) [3, 4]. Type I collagen is approximately 80% of the total collagen in adult human dermis and type III collagen, which predominates in gastrointestinal and vascular connective tissues, represents approximately 10% of the total collagen in the adult human dermis [5, 6]. Although collagen production after injury is normally essential for the process of wound healing, excessive collagen synthesis results in an altered tissue structure and consequently leads to a variety of fibrotic disorders such as hypertrophic scars, keloids, scleroderma/systemic sclerosis, liver cirrhosis, and Crohn’s disease. The initiation and progression stages of fibrosis are affected by several growth factors and cytokines secreted from inflammatory cells. In particular, transforming growth factor (TGF)-β, a major regulator of extracellular matrix (ECM) synthesis, stimulates the proliferation and activation of dermal fibroblasts and plays a crucial role in the pathogenesis of fibrosis and scar remodeling [7–9]. During these processes, Smad 2/3 pathways and the extracellular signal-regulated kinase (ERK)/Ets-1 signaling control scar remodeling and fibrosis [9, 10, 11]. Lipo-prostaglandin E1 (Lipo-PGE1, Alprostadil) is a lipid emulsion of prostaglandin E1 (PGE1) that is used as a vasodilator in the treatment of peripheral vascular diseases. PGE1 accelerates the repair of skin ulcers and limb ischemia. Our recent study demonstrated that Lipo-PGE1 directly facilitates wound healing *in vivo* and increases HDF migration through the enhancement of CXCR4 [12]. Despite the beneficial effects of Lipo-PGE1 during the proliferation/migration phases of wound healing, the regulatory effect of Lipo-PGE1 on collagen synthesis and the underlying detailed mechanism are not well known.

## Materials and methods

### Cell culture

Primary HDFs from adult normal skin (CRL-1947) and neonatal foreskin (CRL-2097) or keloid tissue (CRL-1762) were obtained from the American Type Culture Collection (VA, USA). The cells were maintained in Dulbecco’s modified Eagle’s medium (Thermo Fisher Scientific, Logan, USA) containing 10% heat-inactivated fetal bovine serum (Thermo Fisher Scientific, Logan, USA), 100,000 U/L penicillin, and 100 mg/L streptomycin (Thermo Fisher Scientific). These cells were used for experiments while in their log phase of growth. The cells were incubated in a humidified atmosphere containing 5% CO_2_ at 37°C and were used at passages 4–10.

### Treatment of cells with inhibitors

Cells were seeded at a density of 3 × 10^5^ cells per 6-well plate. After 12 h, the cells were washed with serum-free medium and then cultured overnight in media lacking fetal bovine serum. In some experiments, cells were pretreated with 10 μM PD98059 (Sigma, MO, USA), an ERK inhibitor, or 0.1 μg/mL TGF-β (R&D Systems, MN, USA) for 1 h, before the addition of Lipo-PGE1 (5 ng/mL; Mitsubishi Tanabe Pharma Korea Co., Hwaseong, Korea).

### Real-time PCR

Total RNA was isolated using TRIzol reagent (Life Science, Carlsbad, USA) and quantified using a NanoDrop 2000 instrument (Thermo Fisher Scientific). One microgram of total RNA was used for first-strand cDNA synthesis with M-MLV reverse transcriptase (Promega, WI, USA) according to the manufacturer's instructions. The primers used were as follows: GAPDH (forward, 5’-ATC ACC ATC TTC CAG GAG CGA-3’; reverse, 5’-TTC TCC ATG GTG GTG AAG ACG-3’), collagen 1A1 (COL1A1; forward, 5’-AAG GTG CTG ATG GCT CTC CT-3’; reverse, 5’-CTG GGA CCA CTT TCA CCC TT-3’), collagen 1A2 (COL1A2; forward, 5’-GAA AGA AGG GCT TCG TGG TC-3’; reverse, 5’-CAG CAG TAC CAG CCT CTC CA-3’), and collagen 3A1 (COL3A1; forward, 5’-AGG TCC TGC GGG TAA CAC T-3’; reverse, 5’-ACT TTC ACC CTT GAC ACC CTG-3’). Real-time PCR was performed using the 7900 Real-Time PCR System (Applied Biosystems, CA, USA) with Power SYBR Green Master Mix (Applied Biosystems, Warrington, UK). The relative levels of gene expression were calculated using the expression of GAPDH as an internal standard. The cycling profile for real-time PCR (40 cycles) was as follows: 95°C for 2 min, 95°C for 5 sec, and 60°C for 30 sec. The relative amounts of gene expression were calculated by using the expression of GAPDH as an internal standard. Data are expressed as normalization of ratio (target gene/GAPDH).

### Measurement of total collagen

The Sircol soluble collagen assay (Biocolor Ltd., Carrickfergus UK) was used to quantify total soluble collagen. Briefly, 100 μL of the conditioned medium was mixed with 1 mL of Sircol dye for 30 min and then centrifuged at 12,000 rpm for 10 min to pellet the formed collagen-dye complexes. After removing the supernatant, the pellet was dissolved in 250 μL of alkali reagent and vortexed. Thereafter, 200 μL of each sample was transferred to an individual well of a 96-microwell plate. Relative absorbance was measured at 555 nm.

### Western blotting

Whole cell lysates were prepared by extracting proteins using RIPA buffer containing 50 mM Tris (pH 7.4), 1% NP-40, 150 mM NaCl, 1 mM EDTA, 1 mM PMSF, 1 mM Na_3_VO_4_, 1 mM NaF, 1 g/mL pepstatin, and 1 g/mL aprotinin. Western blot analysis was performed as previously described [13]. The protein concentration was measured with a Bradford assay (Bio-Rad, Hercules, USA), and an equal amount of each lysate was separated on a 10% SDS-polyacrylamide gel. After the proteins were transferred to polyvinylidene fluoride membranes (Millipore, MA, USA), the membranes were blocked for 1 h in TBST containing 5% skimmed milk. Blocked membranes were then incubated with primary antibodies (goat anti-type I collagen (#1310); Southern Biotechnology, Birmingham, AL, anti-phospho-ERK (#4377), anti-ERK (#9102) and anti-Ets-1 (#14069), anti-phospho-Smad2 (#3108), anti-Smad2 (#8685); Cell Signaling Technology, MA, USA,) diluted 1:1000 overnight at 4°C, washed three times with TBST for 15 min, and incubated with horseradish peroxidase-conjugated anti-goat or anti-rabbit secondary antibody (Sigma Aldrich, St. Louis, USA) diluted 1:5000 for 1 h at room temperature. The membranes were visualized using an ECL detection kit (Thermo Fisher Scientific).

### Small interfering RNA (siRNA) transfection

Cells were transfected with Ets-1-targeting small interfering RNA (siRNA) or negative control siRNA (Santa Cruz, CA, USA) using Lipofectamine RNAiMax (Invitrogen, CA, USA). Briefly, when the cells reached 60–70% confluency, siRNA at a final concentration of 100 nM was combined with Lipofectamine RNAiMax and allowed to complex by incubation for 20 min at room temperature. The mixed solution was added to cells in a 6-well plate and incubated at 37°C for 18 h in serum-free Dulbecco’s modified Eagle’s medium. Thereafter, cells were incubated in the presence or absence of 5 ng/mL Lipo-PGE1. Six hours later, cell extracts were prepared and subjected to western blot analysis with an anti-Ets-1 antibody. After 48 h, cells were maintained in complete medium to examine the effect on collagen production.

### Statistical analysis

Statistical significance was estimated using the Student’s *t*-test. Mean differences were considered to be significant when *P*<0.05. The results are shown as the mean ± SD.

## Results

### Lipo-PGE1 suppresses collagen expression in human adult and keloids fibroblasts

**Fig 1:**
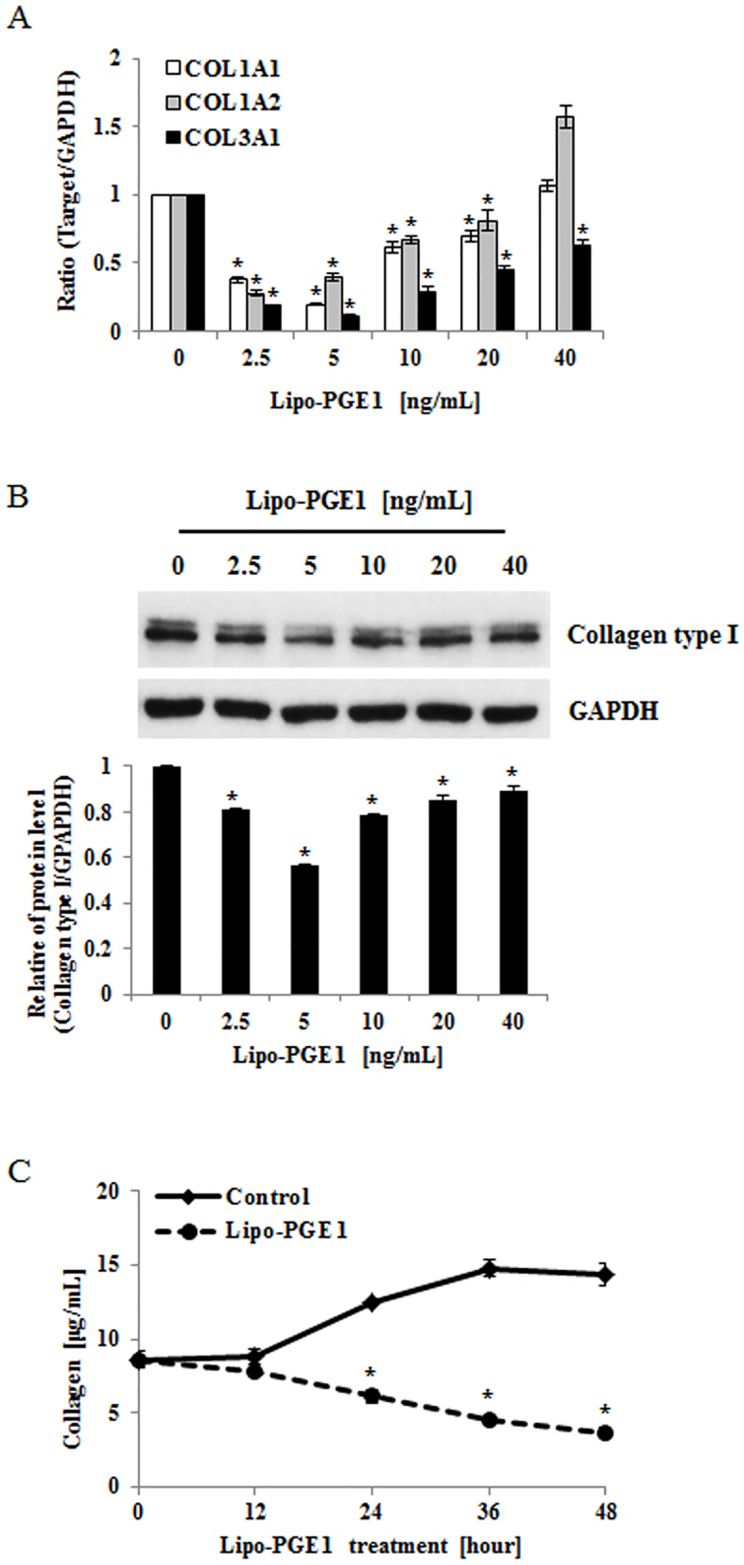
Lipo-PGE1 markedly reduces collagen expression and production in HDFs. (A) HDFs were harvested after Lipo-PGE1 treatment (2.5–40 ng/mL) for 24 h. Total RNA was extracted and cDNA was synthesized for real-time RT-PCR. Bars indicate mean ± SD of three independent experiments, each with triplicate samples. *P<0.05 (B) Effects of Lipo-PGE1 on expression of type I collagen protein in HDFs. Cells were harvested after 36 h and collagen expression was analysed by Western blot using anti-type I collagen antibody. (C) HDFs were harvested after Lipo-PGE1 treatment (5 ng/mL) for the indicated duration. Total collagen was detected by the Sircol assay, which was performed in triplicate. Data are mean ± SD. *P<0.01 *vs*. control at the indicated time point.

**Fig 2:**
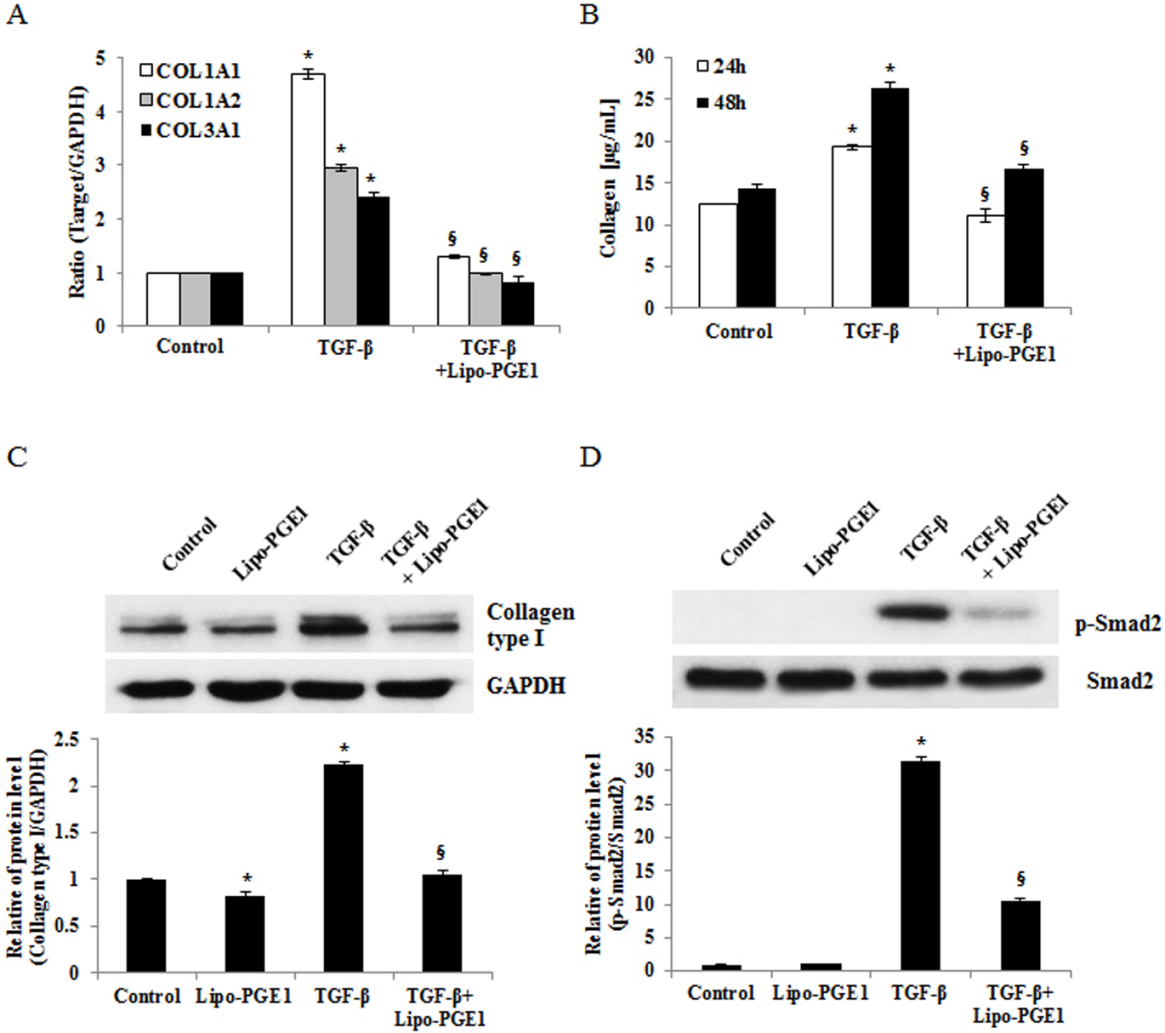
Lipo-PGE1 inhibited collagen gene expression and production induced by TGF- β. (A) HDFs were pretreated with 10 ng/mL TGF-β for 2 h, before addition of 5 ng/mL Lipo-PGE1. After 24 h, total RNA was extracted and real-time RT-PCR was performed. Bars indicate mean ± SD of three independent experiments, each with triplicate samples. *P<0.05 *vs*. control, ^§^*P*<0.05 *vs*. TGF-β-treated group. (B) The total collagen concentration in conditioned medium was determined by the Sircol assay after 48 h of culture. Data are mean ± SD. *P<0.05 *vs*. control, ^§^*P*<0.05 *vs*. TGF-β-treated group. (C) Effects of Lipo-PGE1 on TGF-β-induced type I collagen expression. HDF cells were pretreated with TGF- β (10 ng/ml) for 2 h, and then incubated with 5 ng/mL Lipo-PGE1 for 36 h. Type I collagen production was analyzed by Western blot analysis. (D) Lipo-PGE1 inhibited TGF-β-induced phosphorylation of Smad2. HDF cells were treated with Lipo-PGE1, and then challenged with TGF- β (10 ng/ml). After 36 hours, whole cell lysates were probed with antibodies against Smad2, phospho-Smad2 in Western blots. The band intensities were quantitated and data are mean ± SD. *P<0.01 *vs*. control, ^§^*P*<0.01 *vs*. TGF-β-treated group.

To examine the effect of Lipo-PGE1 on collagen expression in HDFs, cells were treated with various doses (2.5, 5, 10, 20, and 40 ng/ml) of Lipo-PGE1 for 24 h and collagen mRNA expression and cell viability were measured. As shown in Fig 1A, COL1A1 COL1A2, and COL3A1 mRNA synthesis was decreased by all the tested doses of Lipo-PGE1, except 40 ng/mL. Concurrently, there was no toxic effect up to 40 ng/ml of Lipo-PGE1 on HDFs (S1 Fig). To further evaluate the effects of Lipo-PGE1 on types I collagen protein, accounts for around 80% in adult human dermis, Western blot analysis of types I collagen was performed. Type I collagen expression was also decreased by Lipo-PGE1, showing the maximal effect with 5 ng/mL Lipo-PGE1 (Fig 1B). Next, time kinetics were measured by the Sircol collagen assay for 12–48 hours. Total soluble collagen was also decreased by Lipo-PGE1 treatment in a time-dependent manner (Fig 1C). To further examine the effects of Lipo-PGE1 on collagen synthesis in a pro-fibrotic response, its influence on cells stimulated with TGF-β, an important regulator of fibrosis and the pathogenesis of fibrotic disorders, was evaluated. TGF-β treatment induced collagen gene and protein expression in HDFs (Fig 2A and 2B). Lipo-PGE1 significantly suppressed TGF-β-induced COL1A1, COL1A2, and COL3A1 mRNA expression and total soluble collagen level (Fig 2A and 2B). Moreover, Western blot analysis indicated that TGF-β-induced type I collagen expression was also reversed by Lipo-PGE1 treatment (Fig 2C). To clarify whether the downregulation of collagen by Lipo-PGE1 is mediated via TGF-β/Smad signaling pathway, we determined the effects of Lipo-PGE1 on Smad 2/3 phosphorylation. Lipo-PGE1 also inhibited TGF- β induction of Smad2 phosphorylation (Fig 2D). Unfortunately, Smad3 phosphorylation was not detected due to low expression levels on HDFs. In addition, we examined whether Lipo-PGE1 regulates collagen gene expression in primary human fibroblasts derived from keloids. Lipo-PGE1 also decreased COL1A1, COL1A2, and COL3A1 mRNA expression (Fig A in S2 Fig) and the total soluble collagen level (Fig B in S2 Fig) in keloid fibroblasts. These data suggest that Lipo-PGE1 could be involved in negative regulation of collagen expression in human keloid fibroblast as well as normal fibroblast.

### The ERK1/2 pathway is required for the inhibitory effects of Lipo-PGE1 on collagen production

**Fig 3:**
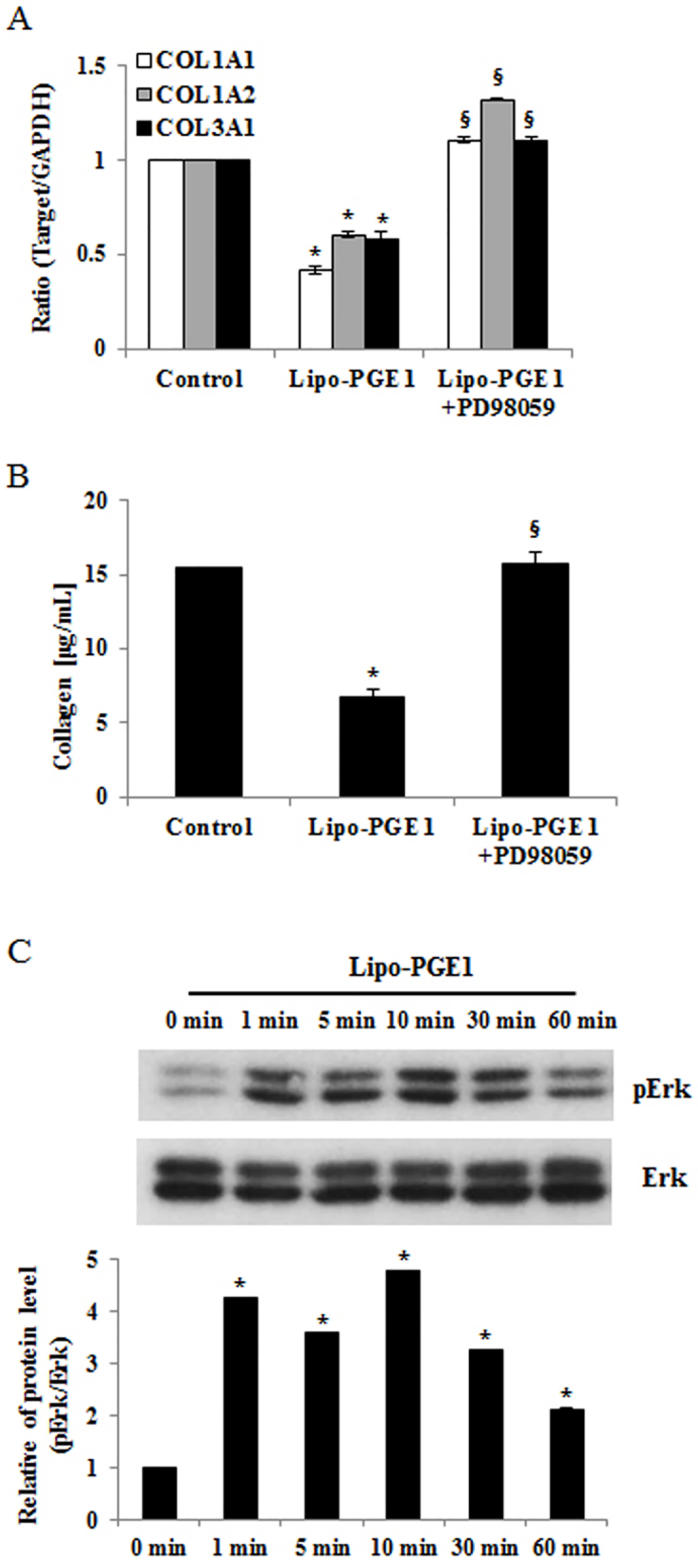
Activation of the ERK pathway is involved in Lipo-PGE1-induced collagen inhibition. (A) HDFs were pretreated with or without a specific inhibitor of ERK1/2 (PD98059) for 1 h and then incubated with 5 ng/mL Lipo-PGE1. Total RNA was extracted and cDNA was synthesized for real-time RT-PCR. Data are mean ± SD of three independent experiments. *P<0.05 *vs*. control; ^§^*P*<0.05 *vs*. Lipo-PGE1-treated group. (B) Cells were pretreated with or without a specific inhibitor of ERK1/2 (PD98059) for 1 h and then treated with 5 ng/mL Lipo-PGE1. Total collagen was determined by the Sircol assay. A representative of three independently performed experiments is shown. *Bars*, mean ± SE. *P<0.05 *vs*. control, ^§^*P*<0.05 *vs*. Lipo-PGE1-treated group. (C) Cells were treated with 5 ng/mL Lipo-PGE1 for 1 min, 5 min, 10 min, 30 min and 60 min. After cell lysis, the level of ERK1/2 phosphorylation was determined by Western blot analysis. The levels of total ERK were used to confirm equal loading of the cell lysates.

ERK1/2 pathway is actively involved in collagen gene expression, and ERK1/2 activation is linked to ECM remodeling [14, 15]. To determine whether ERK signaling pathways are involved in the regulation of Lipo-PGE1-inhibited collagen synthesis, HDFs were pretreated with an inhibitor of ERK (PD98059) before the addition of 5 ng/mL Lipo-PGE1. As expected, PD98059 effectively recovered Lipo-PGE1-inhibited collagen gene and protein production (Fig 3A and 3B). To directly evaluate the activation of ERK, we analyzed its phosphorylation by western blotting. Lipo-PGE1 rapidly induced phosphorylation of ERK within 1 min, with the maximal effect after 10 min (Fig 3C). Together, these results indicate that ERK is involved in the signaling pathway underlying Lipo-PGE1-induced collagen inhibition in HDFs.

### Ets-1 is involved in Lipo-PGE1-mediated collagen inhibition

**Fig 4:**
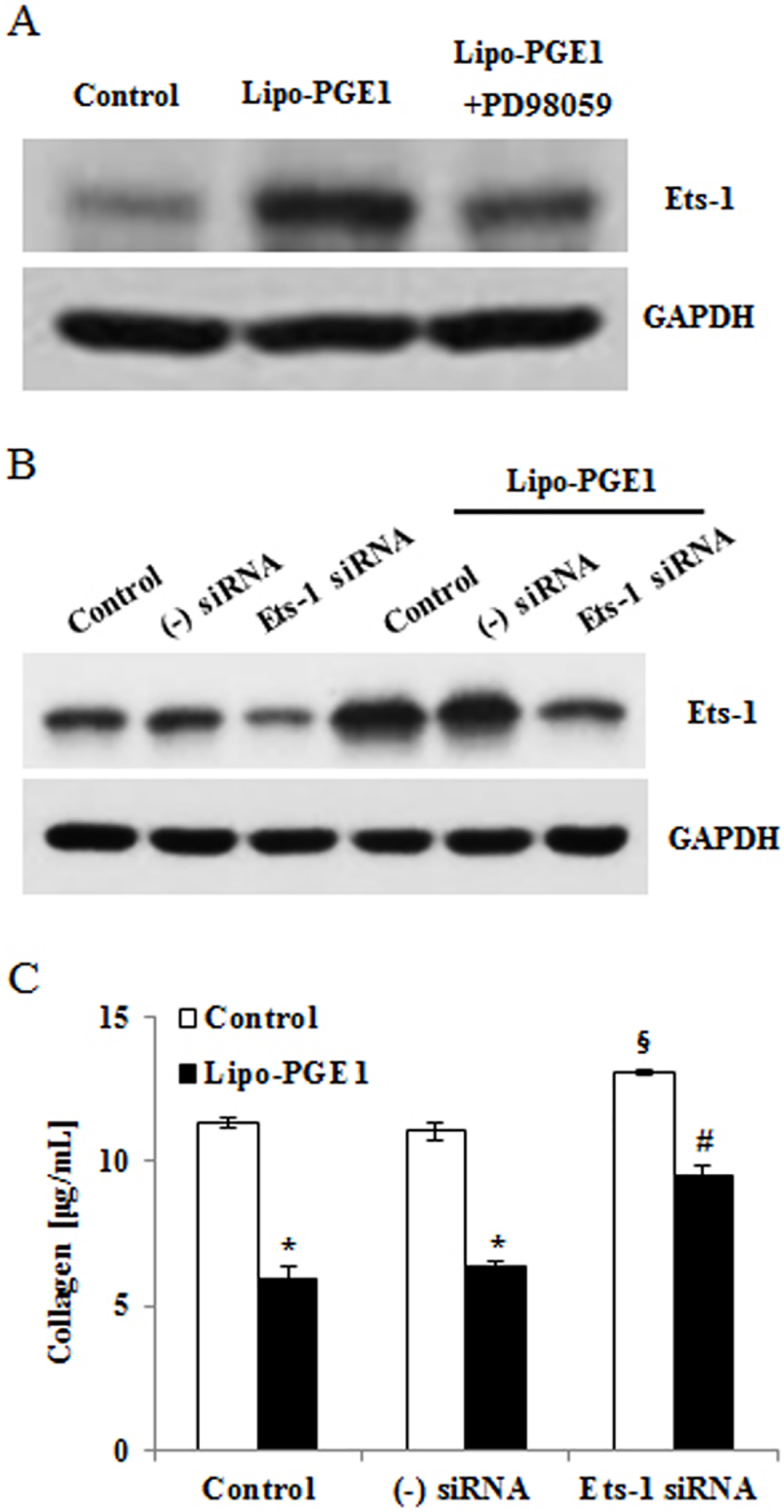
Effects of Ets-1 on Lipo-PGE1-induced collagen inhibition in HDFs. (A) HDFs were pretreated with or without a specific inhibitor of ERK1/2 (PD98059) for 1 h and then treated with 5 ng/mL Lipo-PGE1. Ets-1 protein was confirmed by Ets-1-specific western blot analysis. (B) HDFs were treated with Lipo-PGE1 and transfected with siRNA targeting Ets-1 or a negative control sequence. After stabilization, cells were collected and Ets-1-targeting siRNA transfection was confirmed by Ets-1—specific western blot analysis. (C) Collagen production after Ets-1-targeting siRNA transfection was detected by the Sircol assay. Data are mean ± SD of three independent experiments. *P<0.05 *vs*. control, ^§^*P*<0.05 *vs*. non-treated cells transfected with negative control siRNA, ^#^*P*<0.05 *vs*. Lipo-PGE1-treated cells transfected with negative control siRNA.

Ets-1 was recently shown to be associated with key regulation of collagen deposition and one of the target genes downstream of ERK1/2 [16]. It was reported that regulation of collagen expression by the ERK pathway in dermal fibroblasts involves activation of Ets-1 [14–16]. To further investigate the mechanism underlying Lipo-PGE1-mediated regulation of collagen gene expression, we evaluated the effects of Lipo-PGE1 on the level of Ets-1. As expected, the Ets-1 level was significantly increased in HDFs treated with Lipo-PGE1 for 6 h. Furthermore, in cells treated with PD98059, blockade of ERK1/2 inhibited Lipo-PGE1-enhanced Ets-1 (Fig 4A). We further investigated collagen production after Ets-1-targeting siRNA transfection. Western blot analysis showed that expression of Ets-1 was suppressed by knockdown of Ets-1 (Fig 4B) and treatment with Ets-1-targeting siRNA blocked Lipo-PGE1-induced collagen inhibition (Fig 4C), suggesting a connection between these events. Taken together, these data indicate that Lipo-PGE1 has inhibitory effects on collagen synthesis in HDFs via Ets-1-dependent pathway, suggesting that ERK/Ets-1 are involved in the regulation of collagen synthesis.

## Discussion

The present study showed that Lipo-PGE1 negatively regulates collagen synthesis in HDFs. Our data further showed that Lipo-PGE1 strongly inhibits collagen production via an ERK- and Ets-1-dependent pathway. To our knowledge, this is first report that Lipo-PGE1 has inhibitory effects on collagen synthesis in fibroblasts. According to our previous report, Lipo-PGE1 accelerates wound healing *in vivo* and increases CXCR4-mediated migration of HDFs. In fact, we anticipated that Lipo-PGE1 would increase collagen production; however, unexpectedly, Lipo-PGE1 significantly inhibited collagen synthesis. Interestingly, total soluble collagen was significantly decreased by Lipo-PGE1 treatment in a time-dependent manner whereas Lipo-PGE1 non-treated group naturally and gradually enhances collagen production in HDFs as time goes on (Fig 1C), suggesting potential candidate to treat in scar remodeling and diseases with deregulated collagen turnover. Although Lipo-PGE1 has beneficial effects for the wound healing process, which facilitate *in vivo* wound healing and fibroblast migration, it can also considerably inhibit collagen synthesis. Therefore, it is likely to be appropriate for scar remodeling as well as for modulating the proliferation/migration phases, resulting in direct effects on wound healing in patients. Furthermore, Lipo-PGE1 also suppresses platelet aggregation. Taken together, this suggests that the dosage and timing of Lipo-PGE1 application should be considered for effective enhancement of wound healing and scar remodeling.

The antimicrobial peptide LL-37 has many functions such as enhancing wound repair, angiogenesis, and chemotaxis of neutrophils and monocytes [17–19]. We recently demonstrated that LL-37 suppresses collagen synthesis, suggesting it has an anti-fibrotic function [16]. Therefore, we wondered whether Lipo-PGE1 regulates the LL-37 level, given that they have similar functions such as facilitating wound repair and inhibiting fibrosis in HDFs. Lipo-PGE1 treatment considerably increased LL-37 secretion in human HDFs (Fig A in S3 Fig). In addition, Lipo-PGE1 strongly suppressed the expression of connective tissue growth factor (CTGF), an important mediator of fibrosis and scar formation (Fig B in S3 Fig) [8, 20]. These data suggest that, in addition to its direct effects, other factors and mechanisms can mediate indirect effects of Lipo-PGE1 on collagen synthesis.

According to a recent study by Sobota *et al*., PGE1 induces MAPK signaling [21]. Specifically, Park *et al*. reported that ERK/Ets-1 activation plays a significant role in the inhibition of collagen synthesis signaling [16, 22]. Therefore, the present study examined the involvement of ERK/Ets-1 signaling in Lipo-PGE1-inhibited collagen production in HDFs. The ERK inhibitor PD98059 recovered Lipo-PGE1-induced collagen inhibition in HDFs (Fig 3A and 3B). Treatment with Ets-1-targeting siRNA blocked the downregulation of collagen expression by Lipo-PGE1 (Fig 4B and 4C), demonstrating that ERK/Ets-1 signaling is involved in these processes. Recent evidence suggests that a member of the Ets transcription factor family, Fli1, contributes to key repression of collagen synthesis, notably that of the human COL1A2 gene [23]. Indeed, Lipo-PGE1 increased Fli1 expression, in addition to that of Ets-1 (Fig C in S3 Fig). Thus, the Ets-1 family mediates the inhibition of collagen expression by Lipo-PGE1, suggesting that Lipo-PGE1 has anti-collagenic and anti-fibrogenic effects on HDFs. Further investigations are necessary to clarify the detailed mechanisms underlying Lipo-PGE1-induced collagen regulation in human HDFs and the factors involved.

**Fig 5:**
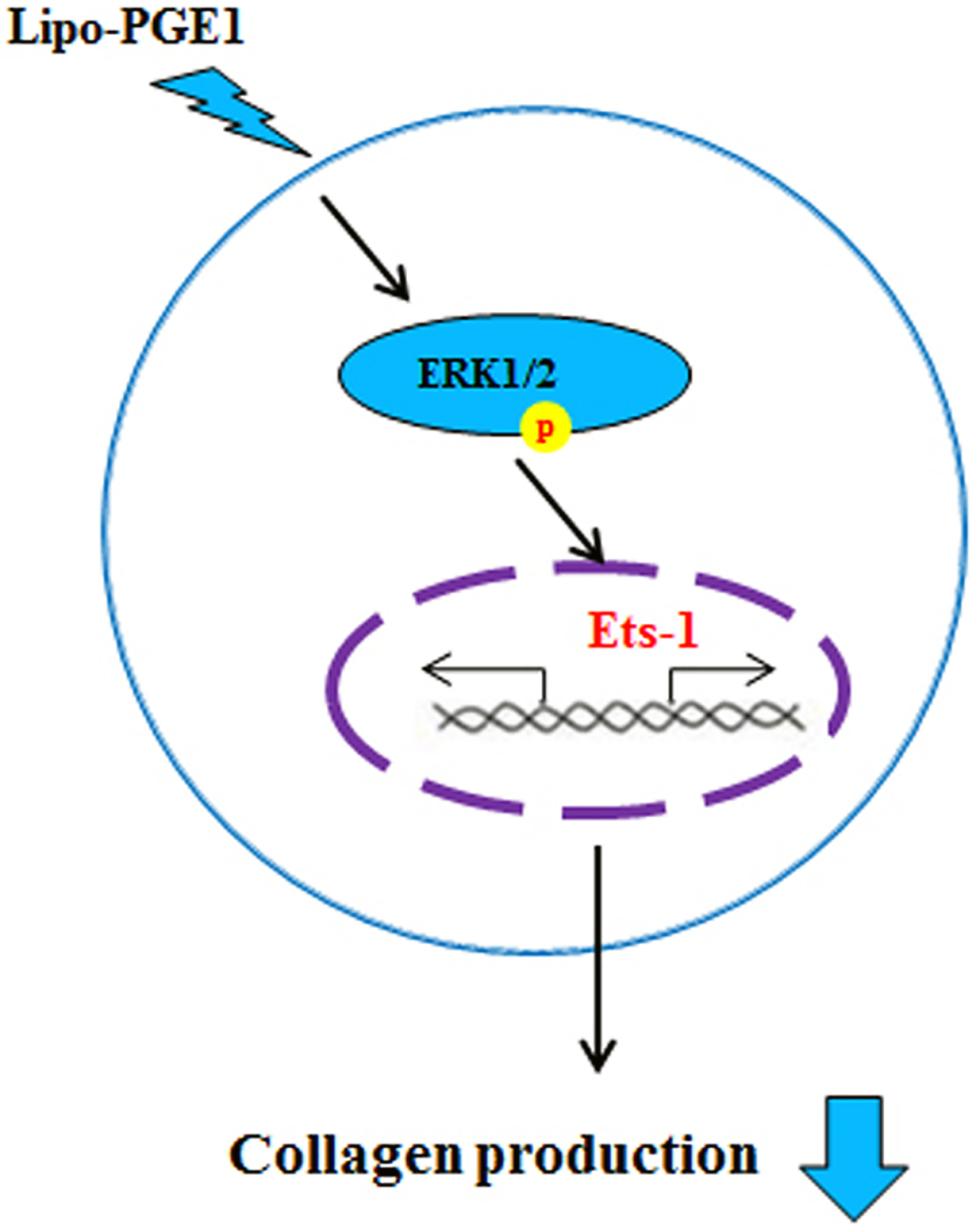
Scheme representing the suppression of collagen production in HDFs after Lipo-PGE1 treatment. Lipo-PGE1 strongly inhibits collagen synthesis in HDFs. Furthermore, an ERK- and Ets-1-dependent pathway is involved in Lipo-PGE1-inhibited collagen production. Lipo-PGE1 can be modulated to down-regulate collagen synthesis in human HDFs and may provide a therapeutic approach to inhibit fibrosis and its roles in the pathogenesis of skin disorders.

In summary, the present study demonstrated the regulatory effect of Lipo-PGE1 on collagen production in HDFs. Additionally, Lipo-PGE1 down-regulates TGF-β-induced collagen. Our data further show that the ERK/Ets-1 pathway is involved in suppression of collagen production by Lipo-PGE1 (Fig 5). To our knowledge, this is the first report that Lipo-PGE1 has inhibitory effects on collagen production in fibroblasts, further suggesting that Lipo-PGE1 is an effective candidate to treat keloids and fibrosis.

## Supporting information

S1 Fig Effects of Lipo-PGE1 on cell viability in HDFs. (DOCX)

S2 Fig Lipo-PGE1 markedly reduces collagen production in keloid fibroblasts. (DOCX)

S3 Fig Lipo-PGE1 markedly reduces the levels of LL-37, CTGF, and Fli1 in HDFs. (DOCX)

## Abbreviations

COL1A1: collagen 1A1
COL1A2: collagen 1A2
COL3A1: collagen 3A1
CTGF: connective tissue growth factor
ECM: extracellular matrix
ERK: extracellular signal-regulated kinase
HDF: human dermal fibroblast
Lipo-PGE1: Lipo-prostaglandin E1
PGE1: prostaglandin E1
siRNA: small interfering RNA
TGF: transforming growth factor
