# Supplementary material for: Lipo-PGE1 suppresses collagen production in human dermal fibroblasts via the ERK/Ets-1 signaling pathway
Source: PLoS One. 2017 Jun 23;12(6):e0179614. doi: 10.1371/journal.pone.0179614 (PMC5482458; doi:10.1371/journal.pone.0179614)
Supplement: S1 Fig — (DOCX) [file pone.0179614.s001.docx]

**S1 Fig**

**
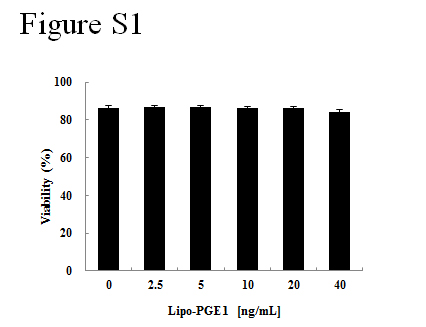
**

**S1 Fig. Effects of Lipo-PGE1 on cell viability in HDFs.** HDFs were treated with the indicated concentrations of Lipo-PGE1 (2.5–40 ng/mL) for 24 h, after which cell viability was measured using a Trypan blue assay, which was performed in triplicate. Results are expressed as percentages of the control.
