# Supplementary material for: Lipo-PGE1 suppresses collagen production in human dermal fibroblasts via the ERK/Ets-1 signaling pathway
Source: PLoS One. 2017 Jun 23;12(6):e0179614. doi: 10.1371/journal.pone.0179614 (PMC5482458; doi:10.1371/journal.pone.0179614)
Supplement: S2 Fig — (DOCX) [file pone.0179614.s002.docx]

**S2 Fig**


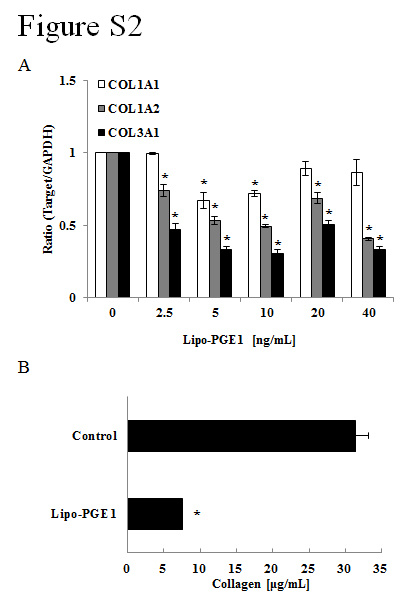


**S2 Fig. Lipo-PGE1 markedly reduces collagen production in keloid fibroblasts.** (A) Keloid fibroblasts were treated with the indicated concentrations of Lipo-PGE1 (2.5–40 ng/mL) for 24 h. Total RNA was extracted and cDNA was synthesized for real-time RT-PCR. Bars indicate mean ± SD of three independent experiments, each with triplicate samples. ^*^*P*<0.05 (B) Keloid fibroblasts were treated with 5 ng/mL Lipo-PGE1 for 24 h. Total collagen was detected by the Sircol assay, which was performed in triplicate. Data are mean ± SD. ^*^*P*<0.05 *vs.* control.
