# Supplementary material for: Lipo-PGE1 suppresses collagen production in human dermal fibroblasts via the ERK/Ets-1 signaling pathway
Source: PLoS One. 2017 Jun 23;12(6):e0179614. doi: 10.1371/journal.pone.0179614 (PMC5482458; doi:10.1371/journal.pone.0179614)
Supplement: S3 Fig — (DOCX) [file pone.0179614.s003.docx]

**S3 Fig**

**
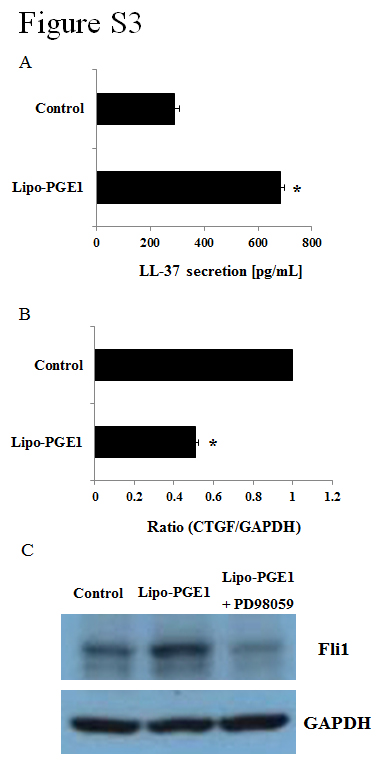
**

**S3 Fig. Lipo-PGE1 markedly reduces the levels of LL-37, CTGF, and Fli1 in HDFs.** (A) HDFs were treated with 5 ng/mL Lipo-PGE1 for 24 h, after which LL-37 production was measured using a LL-37-specific enzyme-linked immunosorbent assay. ^*^*P*<0.05 *vs.* control. (B) HDFs were harvested after Lipo-PGE1 treatment (5 ng/mL) for 12 h. Total RNA was extracted and cDNA was synthesized for real-time RT-PCR analysis of CTGF. Results are mean ± SD of three independent experiments. ^*^*P*<0.01 *vs.* control. (C) Fli1 expression was confirmed by Fli1-specific western blot analysis.
